# Genome-wide and pan-genomic analysis reveals rich variants of NBS-LRR genes in a newly developed wild rice line from *Oryza alta* Swallen

**DOI:** 10.3389/fpls.2024.1345708

**Published:** 2024-04-08

**Authors:** Fimanekeni Ndaitavela Shivute, Yi Zhong, Jinwen Wu, Yueming Bao, Wei Wang, Xiangdong Liu, Zijun Lu, Hang Yu

**Affiliations:** ^1^ State Key Laboratory for Conservation and Utilization of Subtropical Agro-Bioresources, Guangdong Laboratory for Lingnan Modern Agriculture, South China Agricultural University, Guangzhou, China; ^2^ Guangdong Provincial Key Laboratory of Plant Molecular Breeding, South China Agricultural University, Guangzhou, China; ^3^ College of Agriculture, South China Agricultural University, Guangzhou, China; ^4^ Rice Research Institute, Guangdong Academy of Agricultural Sciences, Guangzhou, China

**Keywords:** germplasm, wild rice, *Oryza alta* swallen, genome sequencing, pan-genome

## Abstract

**Introduction:**

*Oryza alta* Swallen is an allotetraploid perennial wild rice and contains CCDD genome, which may harbor favorable genes for the enrichment of genetic resource.

**Methods:**

A new wild rice line, Huaye 5, was developed from *Oryza alta* Swallen in our lab. Whole genome re-sequencing and pan-genomic analysis were employed to analyze its genomic variations and novel genes.

**Results and Discussion:**

More than ten million genomic variations were detected when compared with Asian cultivar. Among the variational genes, 724, 197 and 710 genes coded protein kinase, synthetase and transcription factor, respectively. A total of 353, 131 and 135 variational genes were associated with morphological trait, physiological trait, resistance or tolerance, respectively. A total of 62 were NBS-LRR genes were detected, in which 11 NBS-LRR genes expressed in sheath and mature stem, and 26 expressed in young and mature roots expressed. The pan-genome sequences of wild rice species with CCDD genome were constructed by integrating 8 *Oryza alta* (OA), 2 *Oryza grandiglumis* (OG) and 18 *Oryza latifolia* (OL) accessions. A total of 28 non-reference NBS-LRR genes were revealed, and 7 of which were mainly expressed in mature roots. This research demonstrated rich DNA variation in the *Oryza alta* Swallen that may provide a new germplasm for rice resistance breeding.

## Introduction

Rice (*Oryza sativa* L.) is one of the most important food crops in the world. Approximately half of the world’s population uses rice as a staple food, especially in developing countries (Zhang et al., 2020). Rice improvements help us to meet the challenge of feeding a population by breeding better varieties as fast as we can. However, with the continuous growth of the world’s population and the continuous development of the economy (Zhang et al., 2019), the requirements for the quality of human life have risen sharply. Moreover, decreased relative arable land area (Fei et al., 2018; Xie et al., 2018; Li et al., 2021) and deteriorated ecological environment (Jiang et al., 2021; Zhou et al., 2021) made rice food scarcity prominent (Guo et al., 2022), which attracted attention from all walks of life. Therefore, research on how to improve rice yield, quality, tolerance, and resistance has become an important task for rice breeders (Peng et al., 2009; Kumar et al., 2020; Khan et al., 2021). Germplasm resources, especially wild rice of *Oryza* species, are the basic materials for breeding, which could lead to the next breakthrough in rice breeding (Xiang et al., 2020).

In the long process of evolution, wild rice harbors many favorable genes that are lost during cultivated rice domestication. The history of using the beneficial genes of common wild rice for rice breeding started approximately a century ago. In the 1930s, Ding Ying used Guangdong common wild rice as parents to breed “Zhongshan No. 1”, a productive cultivar with strong cold tolerance and stress resistance (Liu et al., 1998). In the 1970s, Chinese scientists used various ecological types of common wild rice to hybridize with cultivated rice, including rice male sterile lines (Guo et al., 2016). At present, more than 95% of the sterile lines used in the hybrid combination in rice production are of wild abortion or wild abortive cytoplasm (Christian and James, 2019), and more than 20 excellent traits have been identified in wild rice, mainly for disease and insect resistance, stress resistance, and excellent rice quality (Jiang et al., 2004). At the same time, wild rice is used in breeding systems for strong growth advantages (Peng et al., 2021), such as strong tillering ability, fast growth, developed root system, strong regeneration ability, and functional leaf senescence resistance.

*Oryza alta* Swallen (*O. alta*) is an important allo-tetraploid (2n = 48, CCDD) wild rice. *O. alta*, a rare but precious germplasm material for breeding purposes, has many excellent characteristics that have been lost in cultivated rice (Solis et al., 2021; Yu et al., 2021). These wild rice excellent characteristics include resistance to various pests and high biomass yield. Researchers conducted restriction fragment length polymorphism (RFLP) analysis on the hybrid offspring of *O. alta* and cultivated rice and found that the hybrid offspring plants of *O. alta* and cultivated rice have all the genetic materials from both parents with an additional part of the genome great changes (Mao et al., 1995). Wild rice chromosome fragments can “infiltrate” into the cultivated rice genome in a few generations (Zhan et al., 2020). The understanding and research of this possible “infiltration” mechanism helped to transfer beneficial economic traits from the CCDD genome to the AA genome. Recently, a study reported a route of *de novo* domestication of an allotetraploid rice, *O. alta*, that represents the first *de novo* domestication of not only a wild cereal but also polyploid crops with desired traits using precision genome-editing technologies (Yu et al., 2021).

Our previous research developed a new germplasm, Huaye 5, from *O. alta*, and established a protocol for *in vitro* induction of an auto-allotetraploid line (Zhang et al., 2019). Huaye 5 is an important wild rice germplasm harboring many elite genes. Therefore, it needs to study the genome DNA variation and gene expression in the line and auto-allotetraploid rice. The purpose of this study first is to observe the agronomic traits of Huaye 5 and to study its DNA variation compared to cultivated rice using re-sequencing. Our research will find some important elite genes in Huaye 5, which provides a new germplasm for rice resistance breeding.

## Materials and methods

### Investigation of agronomic traits

Agronomic traits were investigated following the guidelines for new plant varieties in the People’s Republic of China. The investigated traits included the following: plant height, number of panicles, panicle length, flag leaf length, flag leaf width, flag leaf length/width ratio, penultimate leaf length, penultimate leaf width, penultimate leaf length/width ratio, antepenultimate leaf length, antepenultimate leaf width, antepenultimate leaf length/width ratio, filled grains, empty grains, total grains, seed setting rate, grain width, grain length, grain length/width ratio, pollen fertility, and grain weight per panicle.

### Genome re-sequencing and detection of genomic variations

Wild rice line Huaye 5 (*O. alta*) was planted in the experimental field of South China Agricultural University, and the cetyl trimethylammonium bromide (CTAB) method was used to extract DNA from the leaves. The qualified DNA samples were built and sequenced according to Illumina Hiseq operation instructions. The raw sequencing data were filtered according to the following conditions: 1) the sequencing adapters were removed and 2) reads in which the percentage of N bases removed exceeds 50% of the read length. Thereafter, the quality of the filtered raw data was tested using FastQC software with default parameters (Andrews, 2010).

BWA (0.7.17-r1188) software was used to map the high-quality sequencing reads that passed the quality check to the MSU7 (Nipponbare, *O. sativa japonica*, AA genome) and PPR1 (*O. alta*, CCDD genome) reference genomes by “BWA-MEM” algorithm with default parameter (Li and Durbin, 2009; Yu et al., 2021). The MarkDuplicates tool in Picard (2.12.1) software was used to remove possible PCR duplicates in the alignment file to improve the accuracy of variant site detection. SAMtools (1.9) software was used to sort and index the SAM files and convert them into BAM files. GATK (Genome Analysis Toolkit, version 3.8-0) software was used to call genomic variations referring to the GATK best practices (McKenna et al., 2010). Based on the gff3 file of the reference genome, variant sites were annotated using SnpEff (4.3s) software with a parameter of “-upDownStreamLen 2000” (Cingolani et al., 2012).

### Functional enrichment analysis of variant genes

The genomic variations between Huaye 5 and the Asian cultivar were first detected by the comparison against the Nipponbare genome. The variant genes were revealed based on the annotation of genomic variations, and they were functionally enriched using the Gene Ontology (GO) and Kyoto Encyclopedia of Genes and Genomes (KEGG) databases. GO enrichment analysis was conducted using agriGO software, and KEGG was enriched using KOBAS (KEGG Orthology Based Annotation System) software.

### RNA sequencing data analysis

Twenty-six RNA sequencing samples including young leaf, young root, young stem, mature leaf, mature root, mature stem, seed, panicle, and sheath of PPR1 were used to illustrate the expression patterns of variant genes, which could be retrieved from the China National Center for Bioinformation with accession number PRJCA002366 (Yu et al., 2021). The transcriptome data were mapped onto the MSU7 (Nipponbare, *O. sativa japonica*) and PPR1 (*O. alta*, CCDD genome) reference genomes using STAR (2.7.1a) software with “–outFilterMultimapNmax 1 –limitBAMsortRAM 16000000000 –outSAMunmapped Within –twopassMode Basic –outSAMtype BAM SortedByCoordinate –quantMode TranscriptomeSAM” parameters (https://github.com/alexdobin/STAR), and the expression matrix was conducted using RSEM software (http://deweylab.github.io/RSEM/) with default parameter. The expression data were illustrated using pheatmap (https://cran.r-project.org/web/packages/pheatmap/).

To calculate the expression level of novel NBS-LRR genes, the assembled non-reference representative (NRR) sequences were used as reference genome, STAR (2.7.1a) software with the same parameters was used to map the sequencing reads to the NRR reference genome, and the expression matrix was conducted using RSEM software with default parameter. The expression data of novel genes were illustrated using pheatmap.

### Population structure analysis

The population structure of wild rice accessions with CCDD genomes was analyzed by principal component analysis (PCA), phylogenetic analysis, and admixture analysis. PCA was conducted using plink software and plotted using imageGP tools (https://www.bic.ac.cn/ImageGP/). Neighbor-joining phylogenetic tree was constructed using an efficient tool VCF2Dis (https://github.com/BGI-shenzhen/VCF2Dis) and illustrated using FigTree (v1.4.3) software (https://github.com/rambaut/figtree). ADMIXTURE (version 1.3.0) software was used to analyze population structure (Liu et al., 2020).

### Construction and annotation of pan-genome sequences

Pan-genome sequences of wild rice accessions with the CCDD genome were constructed using a metagenome-like method. The genome sequencing reads were first mapped onto the PPR1 (*O. alta*, CCDD genome) reference genome using bowtie2 software, and unmapped reads were extracted using samtools software (Langmead and Salzberg, 2012). The unmapped reads were assembled using megahit software to obtain NRR sequences (Li et al., 2015). Potentially contaminated sequences were eliminated by searching the NT database of the National Center for Biotechnology Information (NCBI) using BLAST software, and the sequences belonging to the *Oryza* genus were kept. Sequences were annotated using the EggNOG database (http://eggnog6.embl.de/). Gene presence/absence variations were analyzed by remapping sequencing reads to pan-genome sequences and SGSGeneLoss software (Tay Fernandez et al., 2022).

## Results

### Agronomic trait and resistance evaluation of Huaye 5

**Figure 1:**
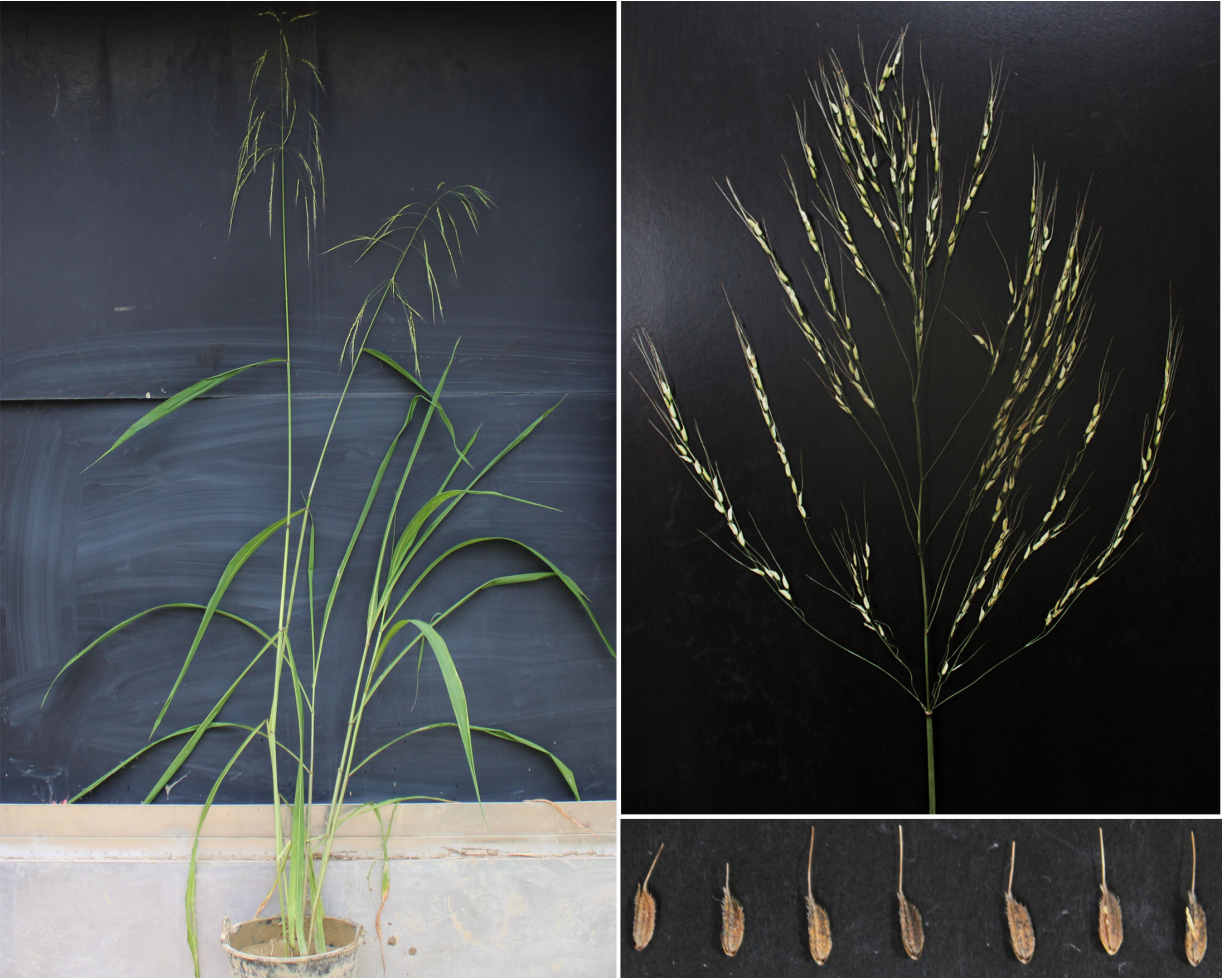
Phenotype of *Oryza alta* wild rice Huaye 5.

Huaye 5 (*O. alta*) plants were planted in our experimental fields, and their agronomic traits were investigated. The plant height of Huaye 5 ranged from 1.97 m to 3.03 m with an average height of 2.39 m, and 46% of the plants were taller than 2.00 m among the tested plants. The panicle number was distributed in the range of 4.00 to 21.00, and more than 74% of total plants obtained 6.00 to 15.00 panicles. The panicle length ranged from 34.50 cm to 113.00 cm with more than 89% ranging from 50.00 cm to 110.00 cm. The length of the flag leaf of Huaye 5 ranged from 31.00 cm to 58.70 cm, and more than 82% ranging from 35.00 cm to 55.50 cm. The width of the flag leaf ranged from 3.60 cm to 6.25 cm, with more than 86% ranging from 3.60 cm to 5.50 cm. The flag leaf length/width (ratio) of Huaye 5 ranged from 5.38 cm to 12.35 cm, and more than 72% of plants were between 7.50 cm and 10.50 cm. Penultimate and antepenultimate leaf length/width ratios were 8.91 and 9.42, respectively. An average of 970.30 grains with an 18.50% seed setting rate and 27.51% pollen fertility were observed in Huaye 5, and its grain length/width ratio was 3.12 (**Figure 1**, **Table 1**).

**Table 1:** Twenty-one main agronomic traits of *Oryza alta* Huaye 5.

No.	Traits	Mean ± SE	Minimum value	Maximum value
1	Plant height (m)	2.39 ± 0.01	1.97	3.03
2	No. of panicles	8.64 ± 0.25	4.00	21.00
3	Panicle length (cm)	67.90 ± 0.82	34.50	113.00
4	Flag leaf length (cm)	41.98 ± 0.27	31.00	58.75
5	Flag leaf width (cm)	4.61 ± 0.02	3.60	6.25
6	Flag leaf length/width (ratio)	9.21 ± 0.09	5.38	12.35
7	Penultimate leaf length (cm)	39.87 ± 0.21	32.30	52.50
8	Penultimate leaf width (cm)	4.53 ± 0.03	3.25	5.85
9	Penultimate leaf length/width (ratio)	8.91 ± 0.08	7.08	13.12
10	Antepenultimate leaf length (cm)	46.57 ± 0.31	34.50	58.00
11	Antepenultimate leaf width	4.99 ± 0.03	3.85	6.25
12	Antepenultimate leaf length/width (ratio)	9.42 ± 0.07	7.22	12.15
13	Filled grains	201.55 ± 13.18	14.00	2,017.50
14	Empty grains	768.75 ± 17.47	235.00	1,700.00
15	Total grains	970.30 ± 24.03	299.50	2,497.50
16	Seed setting rate	18.50 ± 0.40	6.00	76.00
17	Grain width (mm)	2.51 ± 0.01	2.08	2.85
18	Grain length (mm)	7.75 ± 0.03	6.22	8.69
19	Grain length/width (ratio)	3.12 ± 0.02	2.55	3.91
20	Pollen fertility (%)	27.51 ± 21.69	0.25	84.35
21	Grain weight per panicle (g)	2.97 ± 0.97	1.58	4.51

Natural and artificial resistance detection confirmed the insect, drought, and cold resistance in Huaye 5. Moreover, the resistance phenotypes of the wild rice lines were observed for 10 years at our farm, and Huaye 5 showed high-resistance phenotypes to the occurrence of brown planthopper in the epidemic years of 2015 and 2017 and high resistance to other insects, drought, and cold (**Supplementary Figure S1**). *Magnaporthe oryzae* resistance was tested using isolate GUY11 in our lab. Leaves of Huaye 5 (HY5) and Zhonghua 11 (ZH11, cultivated rice CK) were inoculated with *M. oryzae* isolate GUY11, and lesion length was measured after 5 days. Huaye 5 showed significantly smaller lesions than cultivar rice; the lesion length of Huaye 5 was 0.30 mm, 2.59 mm, and 1.60 mm shorter than Zhonghua 11 for replicate experiments 1, 2, and 3, respectively (**Supplementary Figure S2**). With those preliminary results indicating higher blast resistance of wild rice Huaye 5, together with the natural field observations, Huaye 5 could be considered a qualified germplasm for mining NBS-LRR genes.

### Genome re-sequencing and detection of genomic variations in *O. alta*

As the genome heterogeneity for wild rice germplasm, two accessions of Huaye 5 (R01 and R02) were selected for genomic re-sequencing and identification of accordant variations. A total of 47,599,403 and 39,186,507 reads were obtained from R01 and R02, respectively, and 97.71% and 97.41% were high-quality bases (quality score of Q30 level with inferred base call accuracy was 99.9%), respectively. The GC contents for both samples were 42%. The sequencing data were first mapped onto cultivated rice reference genome (MSU7) to reveal homologous variations. A total of 11,207,099 [8,315,629 single-nucleotide polymorphisms (SNPs) and 2,891,470 InDels] and 10,852,375 (8,106,973 SNPs and 2,745,402 InDels) DNA polymorphic sites were identified in R01 and R02 of Huaye 5, respectively (**Supplementary Table S1**).

**Figure 2:**
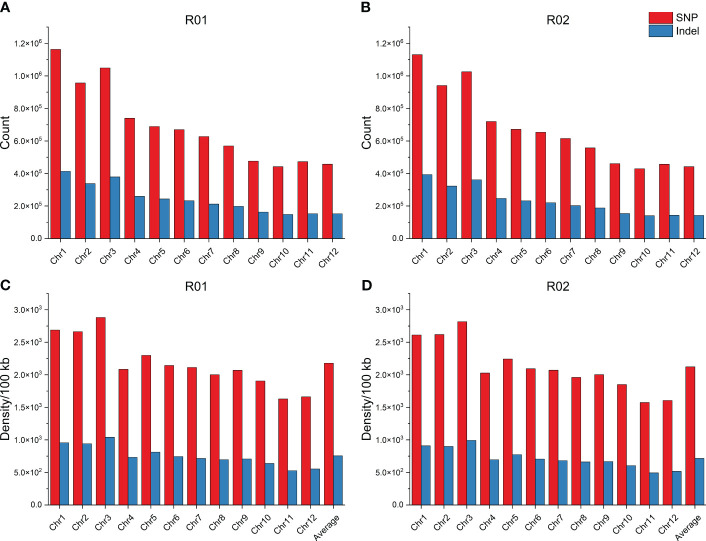
Distribution of genomic variations. **(A)** Distribution of SNP and InDel counts for R01 in each chromosome. **(B)** Distribution of SNP and InDel counts for R02 in each chromosome. **(C)** Distribution of SNP and InDel density for R01 in each chromosome. **(D)** Distribution of SNP and InDel density for R02 in each chromosome. SNP, single-nucleotide polymorphism.

The distribution of variation density and variation number for SNPs and InDels on 12 chromosomes was investigated. The number of SNPs for R01 and R02 was equally distributed, with the highest number of SNPs on Chr1 and the least SNPs on Chr10. Similarly, Chr1 had the highest number of InDels, and Chr10 had the lowest number of InDels. The highest density of SNPs and InDels were found on Chr3, and the lowest were found on Chr11 in the R01 and R02. The average SNP densities were 2,177.35 SNPs/100 kb and 2,122.09 SNPs/100 kb, and the average InDel densities were 754.40 InDels/100 kb and 715.96 InDels/100 kb in R01 and R02, respectively (**Figure 2**).

### Functional enrichment of variant genes

**Figure 3:**
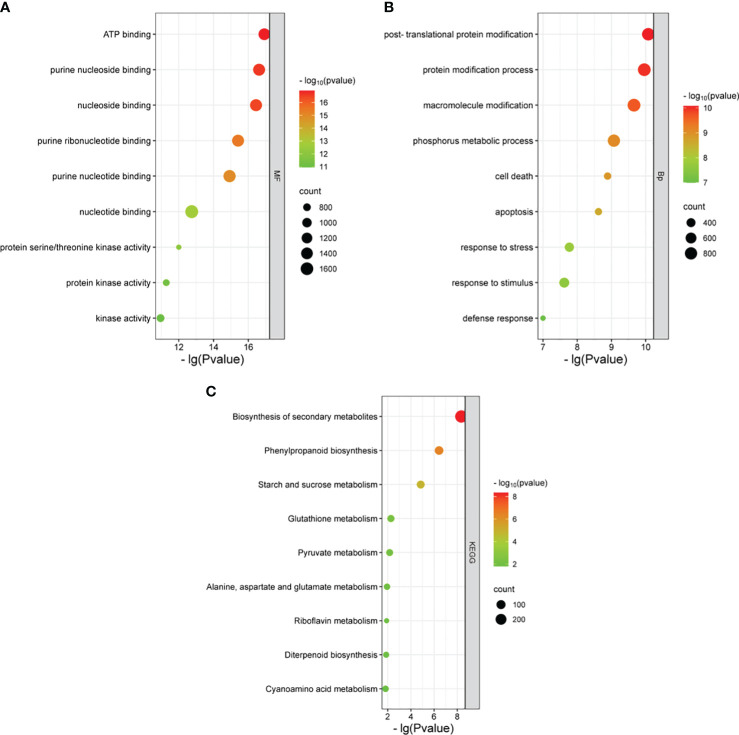
GO and KEGG enrichment for variant genes of Huaye 5. GO enrichment of biological process **(A)**. GO enrichment of molecular function **(B)**. KEGG enrichment **(C)**. GO, Gene Ontology; KEGG, Kyoto Encyclopedia of Genes and Genomes.

Large effect genomic variations (the variations annotated as HIGH and MODERATE by SnpEff software) were considered key variations. A total of 15,993 homozygous variant genes were detected for both R01 and R02 when compared with the Nipponbare reference genome, and GO enrichment analysis showed that these genes were enriched in four biological pathways (cell death, response to stress, macroscopic modification, and phosphorus metabolic process) and three molecular function pathways (ATP binding, protein serine/threonine kinase activity, and nucleoside binding). A total of 420 genes were enriched in response to stress, including 45 NBS-LRR type genes, which were also involved in cell death. Those NBS-LRR genes had 41,113,889 high-effect SNPs and 641,641 high-effect InDels. The KEGG analysis results showed that the 15,994 variant genes were enriched in metabolic pathways like biosynthesis of secondary metabolites, phenylpropanoid biosynthesis, and starch and sucrose metabolism (**Figure 3**).

To identify the genetic difference between Huaye 5 and cultivated rice, we annotated the genetic variations using the annotation of the Nipponbare reference genome. Among the 15,993 variation genes, 724 genes encode protein kinase, 194 genes encode synthase, and 170 genes encode transcription factors. A total of 387 variation genes were known functional genes, including 353 morphological trait-associated genes, 131 physiological trait-associated genes, and 135 resistance- or tolerance-associated genes. The variation genes of resistance or tolerance included 21 bacterial blight resistance genes, four insect resistance genes, 13 cold tolerance genes, 19 drought tolerance genes, 16 salinity tolerance genes, and 62 NBS-LRR genes (**Table 2**).

**Table 2:** Classification of genetic variant genes between Huaye 5 and cultivated rice.

Item	Number	Name of gene
Protein kinase	724	Ser/Thr protein kinase, receptor-like protein kinase 5 precursor, CAMK_CAMK_like_Aur_like.1-CAMK includes calcium/calmodulin-dependent protein kinases, and so on.
Synthase	194	Lachrymatory factor synthase, ATP-citrate synthase subunit 1, GLUCAN SYNTHASE-LIKE protein, starch synthase, and so on.
Transcription factor	170	Histone-like transcription factor and archaeal histone, bZIP transcription factor domain-containing protein, MYB family transcription factor, bHLH transcription factor, and so on.
Morphological trait	353	*GA2ox6*, *SG1*, *OslAA3*, *Os4CL3*, *HDA710*, *OsSuT1*, *RGB1*, *Ostil1*, *OsRMC*, *OsGSR1*, *HDA702*, *OsLIC*, *OsBZR1*, *d2*, *OSH6*, *Ugp1*, *Osjag*, *OsMKP1*, *moc1*, *spp*, and so on.
Physiological trait	131	*OASA2*, *Osg1*, *DCW11*, *OsYSL15*, *ero1*, *OsGLO1*, *rca*, *OsGI*, *OsLFL*, *st1*, *PTC1*, *PAIR2*, *lpa1*, *nol1*, *etr2*, *rsr1*, *DTH8*, *RF1B*, *MAIF1*, *RCN1*, *Badh2*, and so on.
NBS-LRR	62	
Resistance or tolerance	135	
Bacterial blight resistance	21	*RAR1*, *GF14e*, *OSDR8*, *OsRac1*, *OsLOL2*, *OsSS12*, *nls1-1D* *Xa21*, *DEPG1*, *LYP6*, *LYP4*, and so on.
Blast resistance	74	*Pib*, *Pi2*, *Pi9*, *Pb1*, *Pi-ta*, *osbbi1*, *Rir1b*, and so on.
Insect resistance	4	*lsi1*, *Bph14*, *OsHI-LOX*, and *OsPLD*
Cold tolerance	13	*Crr6*, *CDPK13*, *GS1*, *OsTPS1*, *OVP1*, *Osmkk6*, *OsMYB2*, *OsASR1*, *ASR3*, and so on.
Drought tolerance	19	*OsbZIP23*, *OsCAF1B*, *Osdsg1*, *SNAC1*, *OsDIL1*, *OMTN4*, *dsm3*, *OsMIOX*, and so on.
Salinity tolerance	16	*OsGMST1*, *OsKAT1*, *OsCYP2*, *ZFP182*, *OsNHX1*, *OsGAPC3*, *OsCPK21*, and so on.

### Distribution and expression analysis of variant NBS-LRR genes

**Figure 4:**
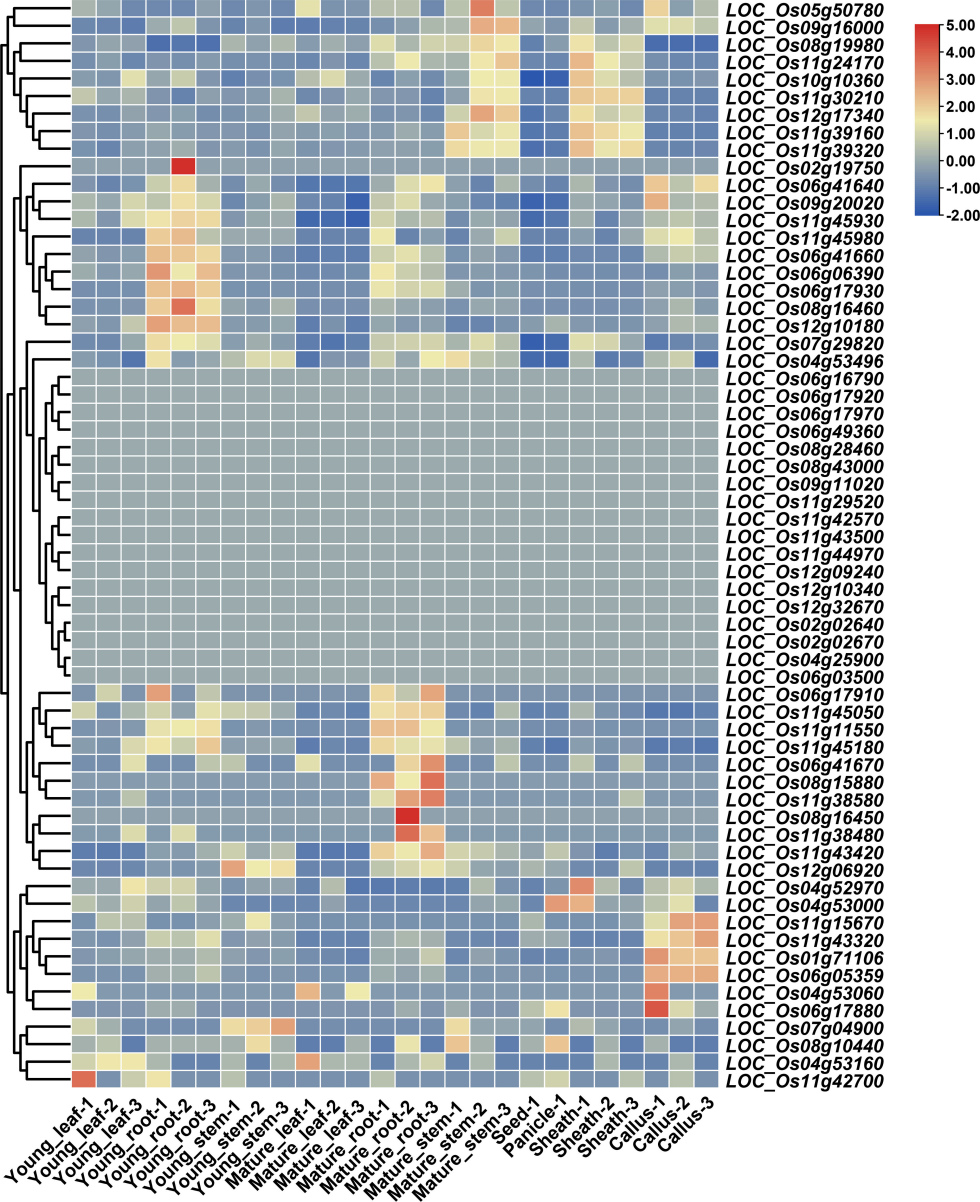
Expression of variant NBS-LRR genes. Values for heatmap of Z-score normalized expression values across samples for each gene.

Gene expression patterns may provide insights into the functional characteristics of the genes. A total of 26 RNA sequencing samples, including three samples for young leaf, three samples for young root, three samples for young stem, three samples for mature leaf, three samples for mature root, three samples for mature stem, seed, and panicle, three samples for sheath, and three samples for callus, were used to explore the expression pattern of the 63 variant NBS-LRR genes. Eighteen variant genes were not expressed in those tissues, 11 genes (*LOC_Os04g52970*, *LOC_Os04g53000*, *LOC_Os08g19980*, *LOC_Os11g24170*, *LOC_Os11g30210*, *LOC_Os10g10360*, *LOC_Os12g17340*, *LOC_Os11g39160*, *LOC_Os11g39320*, *LOC_Os05g50780*, and *LOC_Os09g16000*) were mainly expressed in sheath and mature stem, and 26 genes (*LOC_Os02g19750*, *LOC_Os11g43320*, *LOC_Os12g10180*, *LOC_Os08g16460*, *LOC_Os11g45980*, *LOC_Os06g41640*, *LOC_Os06g06390*, *LOC_Os06g41660*, *LOC_Os06g05359*,*LOC_Os06g17930*, *LOC_Os11g11550*, *LOC_Os04g53496*, *LOC_Os11g43420*, *LOC_Os12g06920*, *LOC_Os07g29820*, *LOC_Os09g20020*, *LOC_Os11g45930*, *LOC_Os06g17910*, *LOC_Os11g45050*, *LOC_Os01g71106*, *LOC_Os11g45180*, *LOC_Os08g16450*, *LOC_Os11g38480*, *LOC_Os06g41670*, *LOC_Os08g15880*, and *LOC_Os11g38580*) were mainly expressed in young and mature root (**Figure 4**).

### Genomic variations and population structure of 30 accessions with CCDD genome

**Figure 5:**
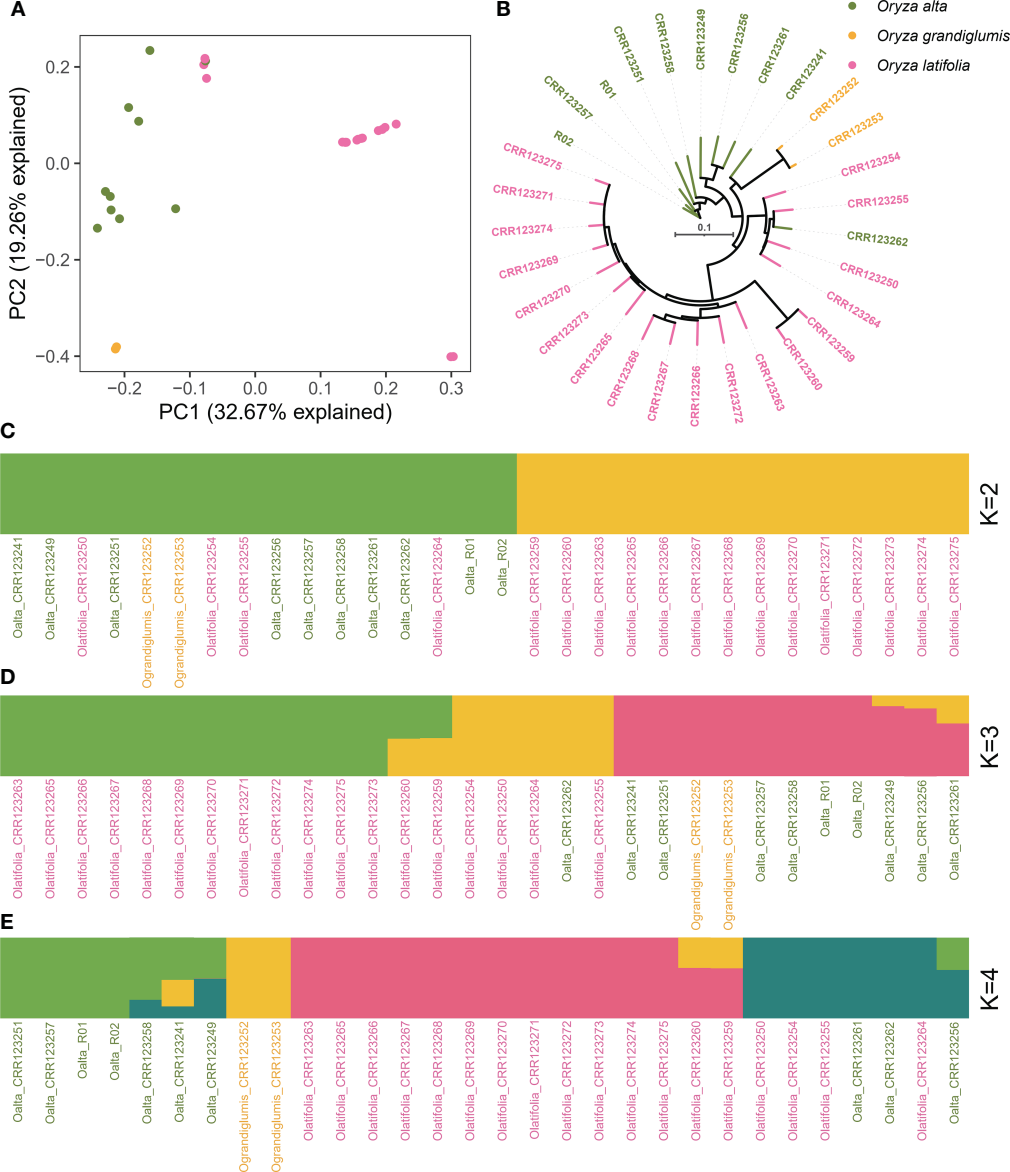
Population structure analysis of 30 accessions with CCDD genome. **(A)** Principal component analysis of 30 wild rice accessions with CCDD genome. **(B)** Phylogenetic tree analysis of 30 wild rice accessions with CCDD genome. **(C–E)** Admixture analysis with different subgroups among 30 wild rice accessions with CCDD genome.

Population structure was analyzed using genomic SNP variations using polyploid rice 1 (PPR1, *O. alta*, CCDD) as the reference genome. Together with Huaye 5 and previously released eight *O. alta* (OA), two *Oryza grandiglumis* (OG), and 18 *Oryza latifolia* (OL) accessions, the population structure of 30 wild rice accessions with CCDD genome were analyzed using PCA, phylogenetic tree, and admixture method (**Figure 5**). PCA results were plotted using the first two principal components, which explained 32.67% and 19.26% variations for PC1 and PC2, respectively (**Figure 5A**). Three species could be classified using PCA (**Figure 5A**) and phylogenetic tree, except for four OL accessions, which were clustered with OA in PCA (**Figure 5B**). Admixture analysis with two subgroups (*k* = 2) clustered 14 OL accessions in a cluster while 10 OA, four OL, and two OG accessions in another cluster (**Figure 5C**). When the subgroup number was set to 3, the same 14 OL accessions were also grouped together, four OL and one OA accessions were in the second group, and nine OA and two OG accessions were in the third group (**Figure 5D**). If the subgroup number was 4, 14 OL accessions were also grouped together, seven OA accessions and two OG accessions were grouped in another two groups, and four OL and three OA accessions were in the last group (**Figure 5E**).

### Assembly and annotation of non-reference representative sequences of the CCDD genome

**Figure 6:**
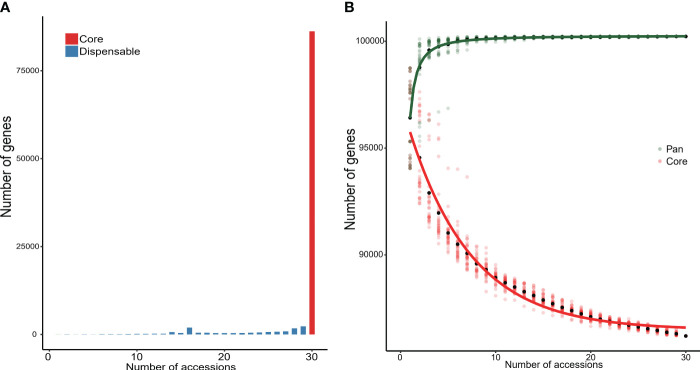
Core and dispensable genes in 30 accessions with CCDD genome. **(A)** Number of core and dispensable genes. **(B)** Number of genes in pan-genome and core-genome of the 30 analyzed genomes.

The 30 wild rice accessions with the CCDD genome were used to detect NRR sequences using a metagenome-like pan-genome analyzing strategy with the PPR1 genome as the reference. A total of 8.39-Mb NRR sequences were assembled with lengths longer than 10 kb; the average length and N50 for assembled contigs were 12.84 kb and 12.53 kb, respectively. Genes in those sequences were annotated, and 926 genes were predicted as novel genes in NRR sequences. Gene presence and absence variations (PAVs) were detected by remapping sequencing reads to the pan-genome. Genes that presented in all accessions were considered core genes, and genes that presented in partial accessions were considered dispensable genes. The number of genes was augmented with the addition of accessions, the final pan-genome gene size was 100,226, the number of dispensable genes was decreased with the addition of accessions, and the final core-genome gene size was 86,204 (**Figure 6**).

**Figure 7:**
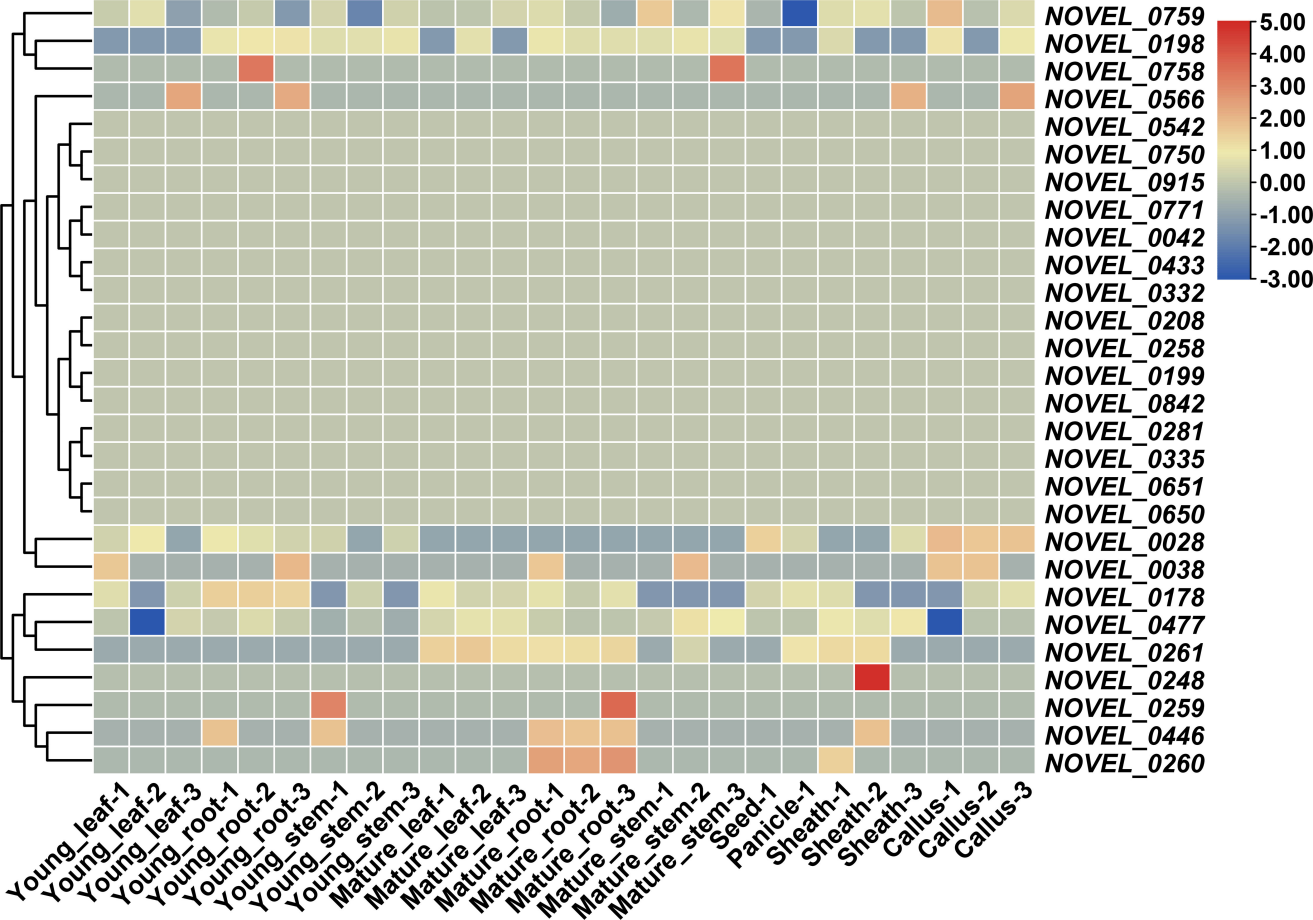
Expression pattern of novel NBS-LRR genes in accessions of CCDD genome. Values for heatmap of Z-score normalized expression values across samples for each gene.

Novel genes were annotated using the EggNOG database, and the NBS-LRR genes were detected using the NB-ARC domain. A total of 28 non-reference NBS-LRR genes were revealed, and three of them were experimentally validated by PCR amplification and Sanger sequencing (**Supplementary Figures S3–S5**, **Supplementary Table S2**). Their expression patterns were analyzed using transcriptome data from the 26 samples. Thirteen genes were expressed in 23 RNA sequencing samples, including seven genes (*NOVEL_0759*, *NOVEL_0198*, *NOVEL_0028*, *NOVEL_0038*, *NOVEL_0178*, *NOVEL_0477*, and *NOVEL_0261*), which have potential constitutive expression in all tissues, and two genes (*NOVEL_0446* and *NOVEL_0260*), which were mainly expressed in mature roots (**Figure 7**). Expression patterns of those novel genes imply their molecular function for further research.

## Discussion

Identification of favorable genetic resources in wild relatives is crucial for the genetic improvement of modern cultivated rice. We previously identified abundant genetic variations in the common wild rice line Huaye 3 (*Oryza rufipogon*, AA genome), which is the progenitor of Asian cultivated rice (Yu et al., 2018). This study released a new *O. alta* wild rice of Huaye 5 and focused on investigating genetic variations in wild rice lines of the CCDD genome and their functional enrichment and expression patterns, which may help their usage in rice breeding and their functional research.

Genome sequencing technology has been applied to various wild rice lines to explore their rich genetic diversity (Xu et al., 2012; Stein et al., 2018; Zhang et al., 2022). In the current study, genome re-sequencing of *O. alta* was conducted, and various genomic variations were revealed. Genome re-sequencing provides valuable information about the genetic diversity present within a species. Our results revealed a significant number of SNPs, insertions, and deletions in the *O. alta* genome. These variations are essential resources for further genetic and functional studies, as they can contribute to the understanding of the genetic basis of important agronomic traits.

The construction of a pan-genome of *Oryza* species can reveal complex genomic diversity and novel hidden genes. A pan-genome for common wild rice and Asian cultivated rice was recently constructed (Stein et al., 2018; Qin et al., 2021; Shang et al., 2022). Here, a total of 30 accessions with the CCDD genome were integrated to reveal the genomic variations and population structure. Furthermore, we performed the assembly and annotation of NRR sequences of the CCDD genome. Non-reference sequences are valuable resources for studying genomic variations and gene functions. Our assembly and annotation efforts resulted in the identification of numerous NRR sequences, providing a valuable resource for future genetic and functional studies in rice.

NBS-LRR genes play a crucial role in plant defense against pathogens. The diversity of those genes in common wild rice lines was revealed in our former study (Yu et al., 2018). We further investigated the distribution and expression patterns of variant NBS-LRR genes in *O. alta* using our newly constructed pan-genome sequences. Our analysis revealed a diverse distribution pattern of these genes across the *O. alta* genome. We also revealed the expression patterns of these genes, indicating their potential usages in functional research and rice breeding. These findings provide valuable insights into the genetic basis of disease resistance in wild rice species.

Taken together, our study provides comprehensive insights into the agronomic traits, genomic variations, functional enrichment, distribution, and expression analysis of variant genes in *O. alta*, as well as the population structure of CCDD wild rice. The findings from this study contribute to our understanding of the genetic basis of important agronomic traits and disease resistance mechanisms in wild rice species. These findings also provide valuable resources for future genetic and functional studies, as well as rice breeding programs aimed at developing improved rice varieties with enhanced stress tolerance and disease resistance.

## Conclusion

In conclusion, the genetic variations of a newly developed *O. alta* Swallen wild rice line, Huaye 5, were investigated, and the rich genomic variations were detected when compared with the Asian cultivar. The integrated analysis with other *O. alta*, *O. grandiglumis*, and *O. latifolia* accessions reconstructed the phylogenetic relationship, and the non-reference sequences were assembled. Variant and non-reference NBS-LRR genes were revealed, and their expression patterns were analyzed. Those results may provide valuable genetic resources for rice molecular breeding and gene functional research.

## Data availability statement 

The raw reads of whole-genome resequencing were deposited in at the NCBI database with accession ID PRJNA1044455. RNA sequencing data could be retrieved in China National Center for Bioinformatics with accession number of PRJCA002366. The sequences and annotations of rice japonica reference genome MSU7 are available from the website https://rice.plantbiology.msu.edu/. All data supporting the conclusions described here are provided in tables, figures, and additional files.

## Author contributions

FS: Writing – original draft, Writing – review & editing. YZ: Investigation, Methodology, Writing – original draft. JW: Methodology, Writing – review & editing. YB: Methodology, Writing – review & editing. WW: Formal analysis, Writing – review & editing. XL: Resources, Supervision, Writing – review & editing. ZL: Formal analysis, Funding acquisition, Investigation, Writing – original draft, Writing – review & editing. HY: Formal analysis, Methodology, Software, Visualization, Writing – original draft, Writing – review & editing.
