# Supplementary material for: Genome-wide and pan-genomic analysis reveals rich variants of NBS-LRR genes in a newly developed wild rice line from Oryza alta Swallen
Source: Front Plant Sci. 2024 Apr 8;15:1345708. doi: 10.3389/fpls.2024.1345708 (PMC11033514; doi:10.3389/fpls.2024.1345708)
Supplement: Supplementary file 1 [file DataSheet_1.docx]

Table S1 Quantity and quality sequencing reads and genomic variations of two samples for Huaye 5.

| Sample | Reads number | Rate of Q30 reads | GC contents | No. of SNPs | No. of InDels | No. of total variations |
| --- | --- | --- | --- | --- | --- | --- |
| R01 | 47,599,403 | 97.71% | 42% | 8,315,629 | 2,891,470 | 11,207,099 |
| R02 | 39,186,507 | 97.41% | 42% | 8,106,973 | 2,745,402 | 10,852,375 |

Table S2 Primers that used to validate three novel NBS-LRR genes.

| Novel genes | Primers |
| --- | --- |
| NOVEL_0042-RA | N0042-1F: ATCCGGTCTCGATAGCTGCAG |
|  | N0042-1R: TTTCGGGAATGTGCTGGATC |
|  | N0042-2R: GACGTTCCAACATTCCATATTCAC |
| NOVEL_0260-RA | N0260-1F: ATGGCAGAAGGCATCGTTG |
|  | N0260-1R: TCTCATTTTCCGGAGCCTCTG |
|  | N0260-2R: CTTCCCAATGATTCCGGTAGAG |
| NOVEL_0261-RA | N0261-1F: CAGAGGGTGTTGTGGCATTG |
|  | N0261-2F: TTTGGGCTGTGGATGTTTGG |
|  | N0261-1R: CGGGTGCCATGAATACTGATC |
