# Supplementary material for: Genome-wide and pan-genomic analysis reveals rich variants of NBS-LRR genes in a newly developed wild rice line from Oryza alta Swallen
Source: Front Plant Sci. 2024 Apr 8;15:1345708. doi: 10.3389/fpls.2024.1345708 (PMC11033514; doi:10.3389/fpls.2024.1345708)
Supplement: Supplementary file 2 [file DataSheet_2.docx]

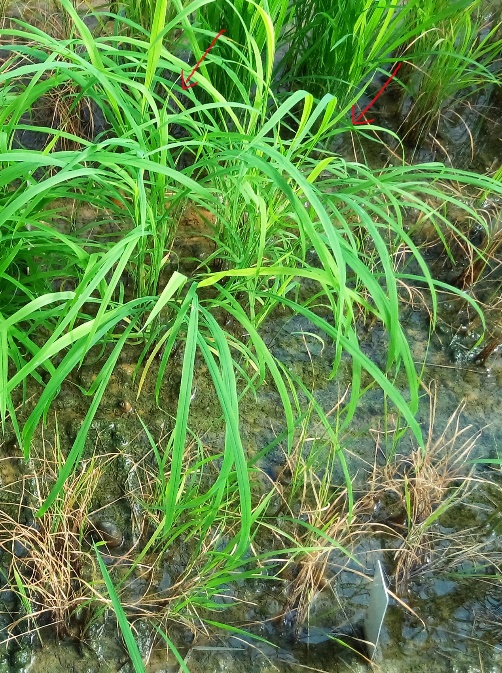


Figure S1 Preliminary resistance evaluation of Huaye 5 (red arrow) to *Magnaporthe oryzae*.


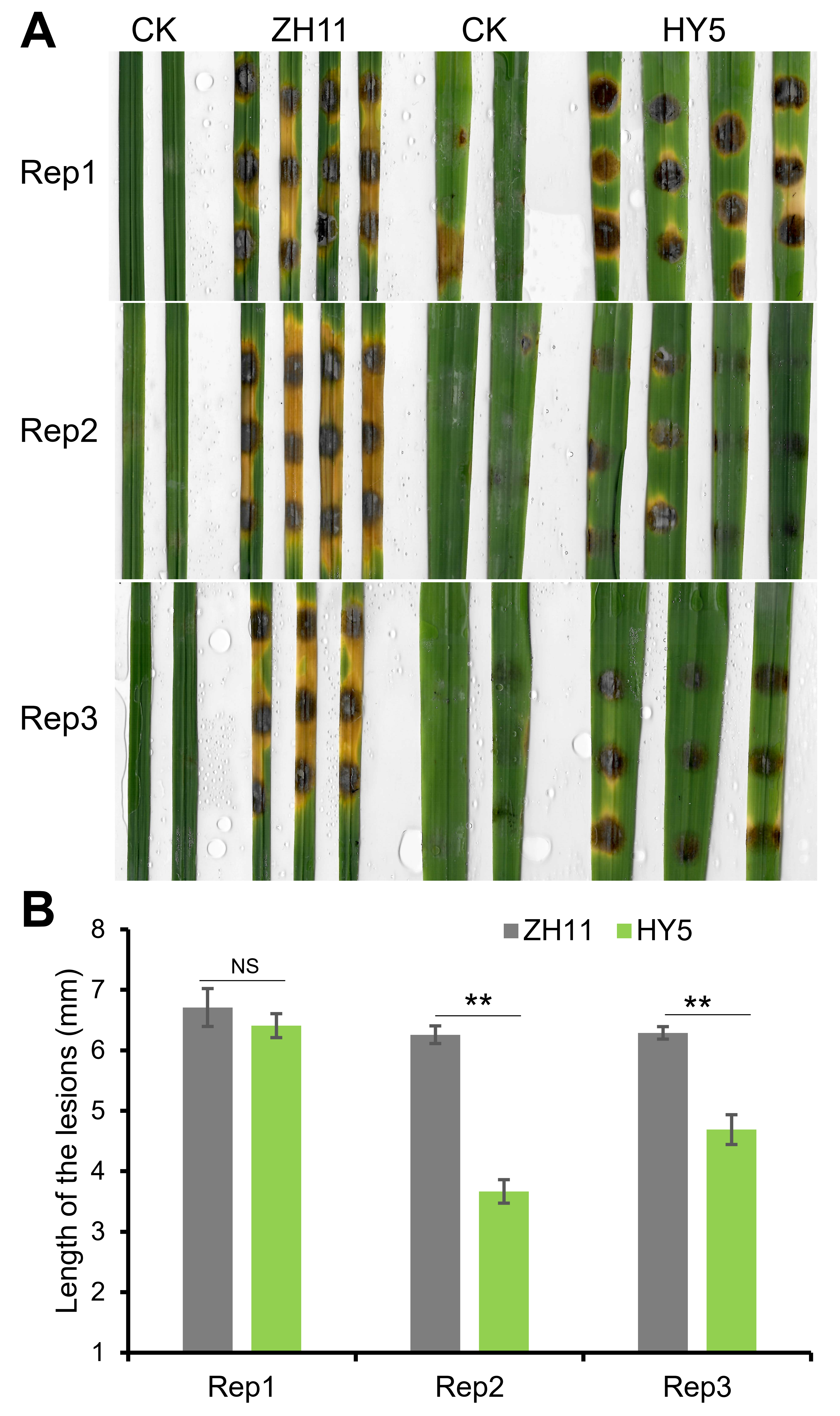


Figure S2 *Magnaporthe oryzae* resistance test using isolate GUY11 in Huaye 5 (HY5).


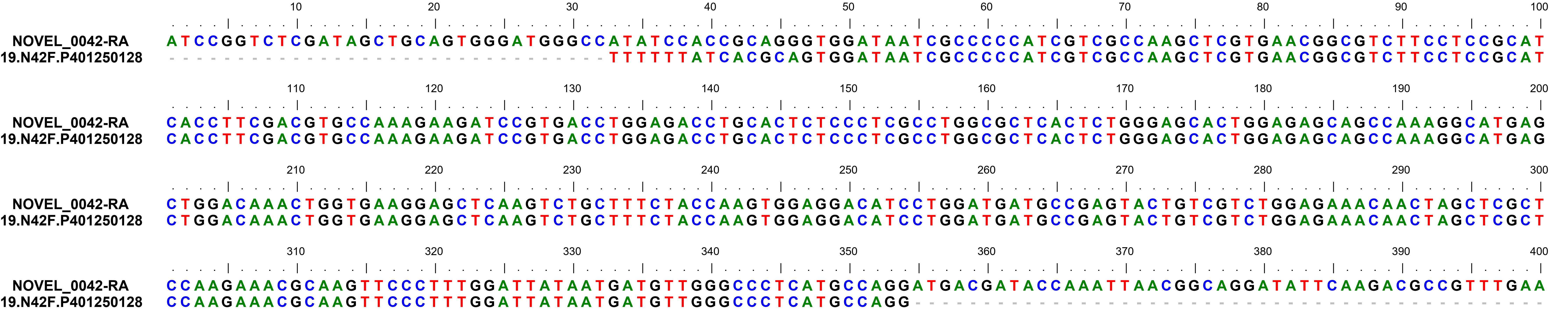


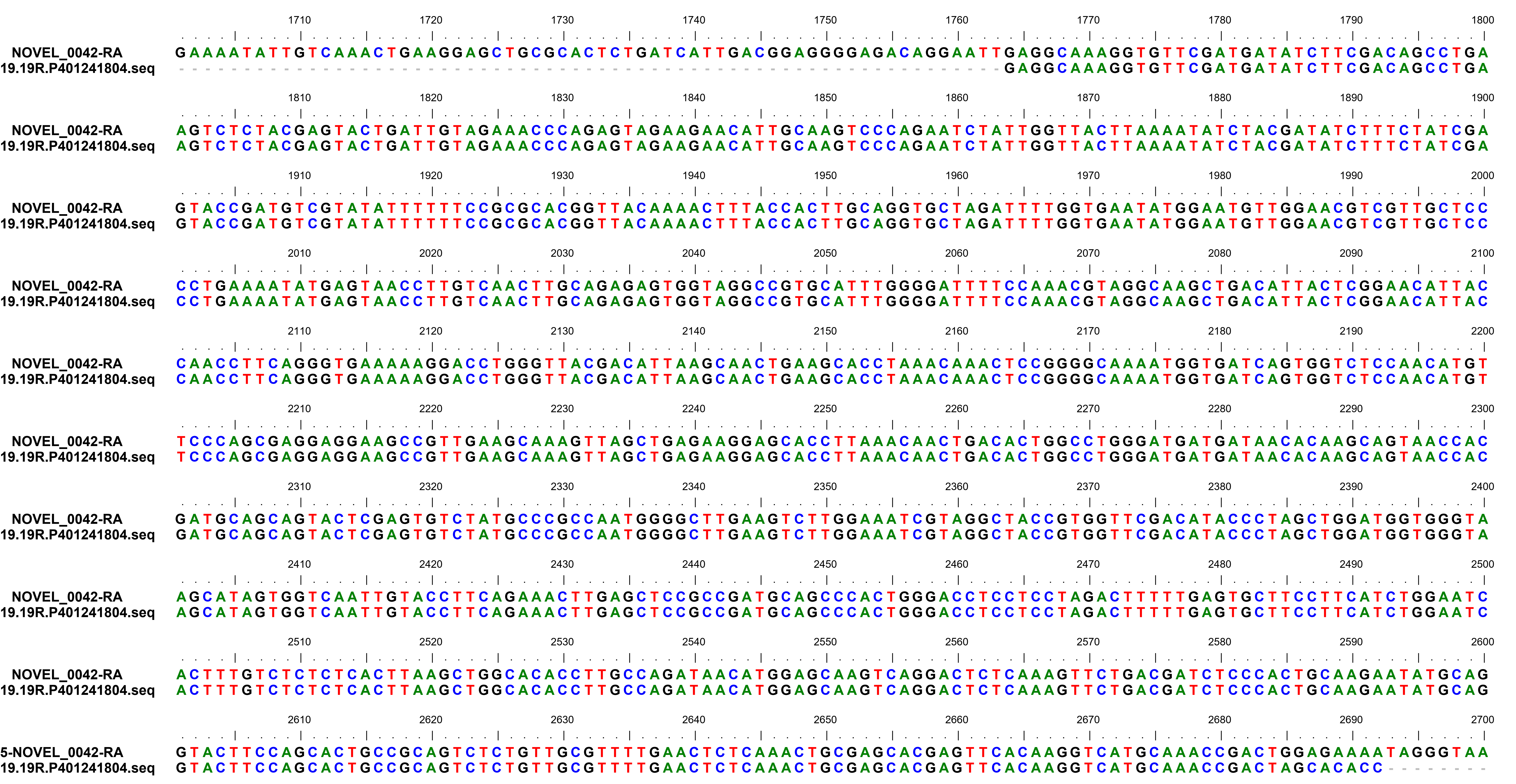


Figure S3 Sequence validation of novel gene *NOVEL_0042-RA* by PCR amplification and Sanger sequencing. The below sequences were the validated sequencing results by Sanger sequencing technology.


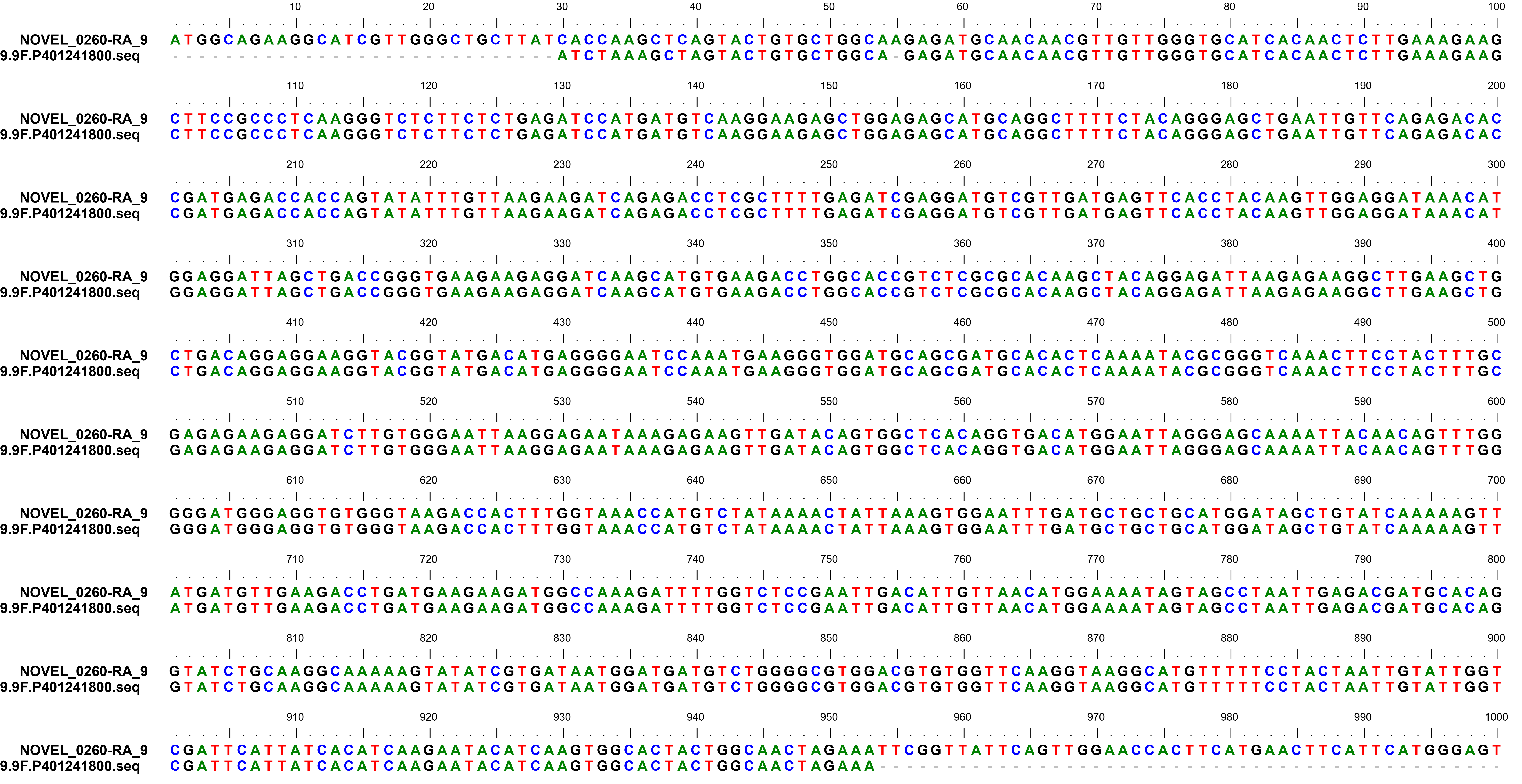


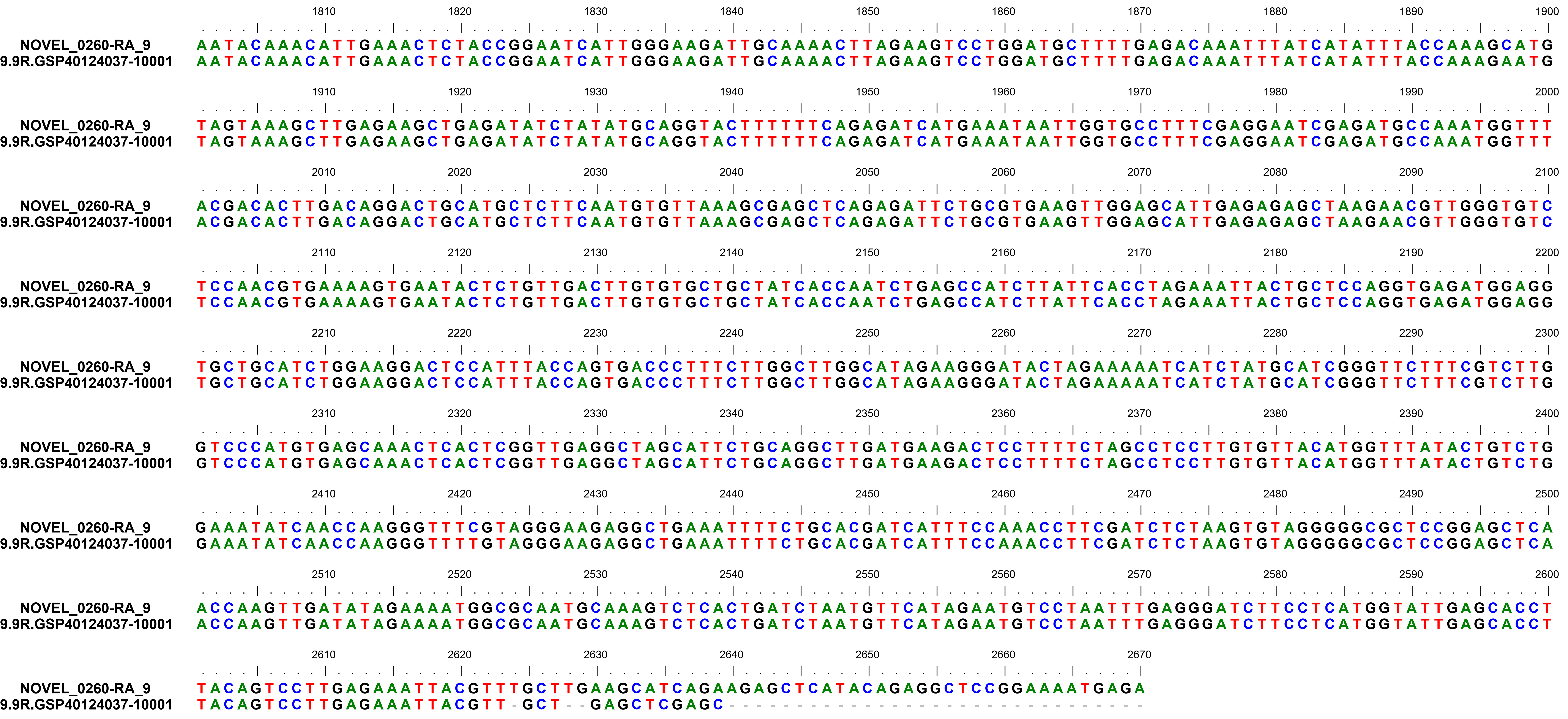


Figure S4 Sequence validation of novel gene *NOVEL_0260-RA* by PCR amplification and Sanger sequencing. The below sequences were the validated sequencing results by Sanger sequencing technology.


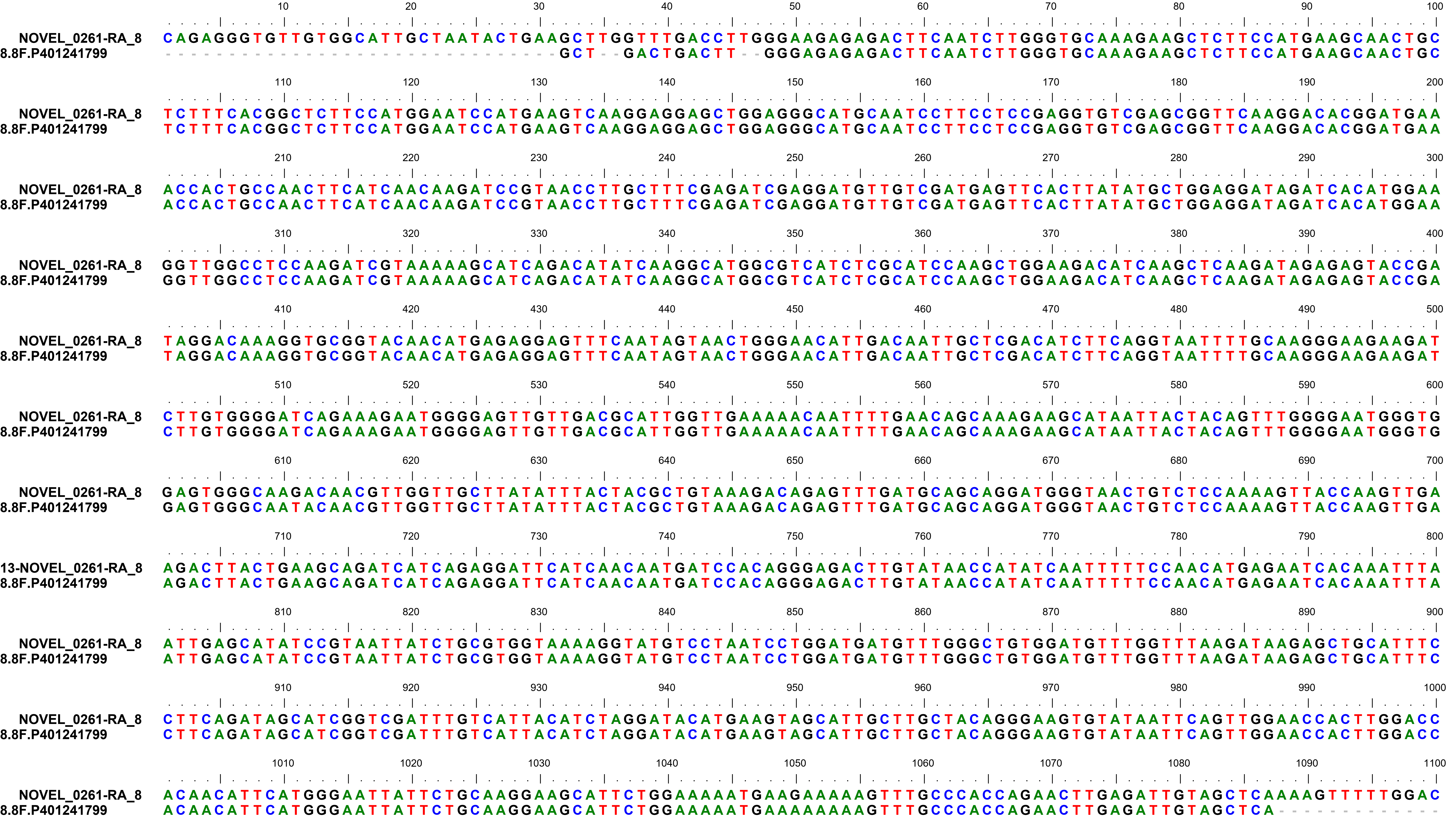


Figure S5 Sequence validation of novel gene *NOVEL_0261-RA* by PCR amplification and Sanger sequencing. The below sequences were the validated sequencing results by Sanger sequencing technology.
